# Bank capital buffer releases, public guarantee programs, and dividend bans in COVID-19 Europe: an appraisal

**DOI:** 10.1007/s10657-022-09734-9

**Published:** 2022-07-18

**Authors:** Alexandra Matyunina, Steven Ongena

**Affiliations:** 1 Swiss Finance Institute, University of Zurich, Plattenstrasse 14, CH-8032 Zurich, Switzerland; 2 Swiss Finance Institute, KU Leuven, NTNU, and CEPR, University of Zurich, Plattenstrasse 14, CH-8032 Zurich, Switzerland

**Keywords:** COVID-19, Regulatory decisions, Regulatory uncertainty, Capital buffer release, Dividend bans, Guarantee schemes, K23, G21

## Abstract

We analyse the recent policy decisions made by the European Central Bank and the national authorities related to capital and shareholders’ remuneration aimed at promoting banking credit supply in COVID-19-afflicted economies. We forecast the impact of the regulatory decisions based on the empirical literature and discuss the factors that reduce the banks’ incentives to expand their loan portfolios. We argue that the introduction of the dividend ban caused a surge in regulatory uncertainty and undermined banks’ market valuation raising the expected funding costs and contributing to the banks’ reluctance to make use of the capital buffers. We develop policy suggestions intended to mitigate this effect.

## Introduction

At the onset of the corona crisis, the European Central Bank (ECB) and the national competent authorities acted promptly to remove regulatory and liquidity constraints for credit supply to the real economy. One of the key measures undertaken was the release of bank capital buffers and communication to the credit institutions that they should make use of the capital requirements relaxation to extend their loan portfolios’ volume and satisfy the economy’s increased liquidity demand. This is the first broad test of the countercyclical capital release mechanism since the idea was introduced in the aftermath of the global financial crisis (GFC).

In this article, we discuss the extent to which the capital related decisions can be effective in the current unprecedented economic environment. Leaning on the recent empirical literature, we estimate the range for the expected aggregate credit growth and give an overview of the factors that can limit the impact of the regulatory efforts.

We start by characterising the borrowing conditions for the euro area companies at the onset of the pandemic in the first half of 2020 based on the available data. The statistics indicate a surge in demand for loans, particularly short-term ones, from firms of all sizes. The banks responded by reducing the loan application rejection rate and easing credit standards for short-term loans. However, both the banks and the firms communicated that the borrowing conditions remained favourable largely due to the public guarantee schemes (PGS). The banks report that credit risk will become the main factor limiting the credit supply after the guarantee programs expire.

We supplement these observations with an evaluation of the effect of country-specific size of PGS relative to a country’s bank sector size on bank lending during 2020 in a cross-section of the euro area banks. The results are consistent with the guarantee programs being efficient in promoting credit growth. Since loans backed by government guarantees imply little to no risk for a bank, larger PGS are associated with a decrease in the riskiness of banks’ assets. As a result, sizable PGS, while stimulating credit supply, reduce the banks’ need for dipping into the regulatory capital buffers made available to them by the ECB.

Further, after providing a concise overview of the current bank capital regulation, we summarise the measures undertaken by the ECB to sustain smooth credit supply to the real economy. We argue that the measures should have the intended effect. Namely, the temporary reduction in the capital requirements has eliminated or mitigated the banks’ incentive to deleverage in the middle of economic turmoil and ensured higher credit supply than would have been obtained in the absence of the respective regulatory actions. This, however, does not guarantee that the increase in aggregate liquidity demand is fully met. The competent authorities have alleviated the capital-related concerns of the banks’ lending decision-making while other constraints related to market discipline remain in place.

Based on the recent studies that focus on the sample of credit institutions similar to the banks that the ECB supervises directly, we forecast the range for the credit supply growth of 1.2–1.5% per 1 percentage point (pp) reduction in capital requirements. For the actual 1.7-pp capital release activated by the ECB and the national authorities at the end of the first quarter this year,^1^ it translates into 2.0–2.6% growth in lending to the real economy over the following 12 months. The magnitude of the impact will increase if the losses and loan delinquencies start accumulating on the banks’ balance sheets as it will allow them to maintain the normal business operation despite the decrease in capital ratios.

The dominant factors that limit the credit supply are the banks’ concerns about their stock market valuation and the extreme uncertainty prevailing in all the elements of the business environment. The euro area banking sector lost 35% of its market value during March 2020, and the unexpected suspension of dividends has contributed to the banks’ prices volatility. As there was no precedent or regulation alerting investors to the fact that their dividend cash flow can be redirected towards the banks’ portfolio expansion in the face of an economic downturn, they are now pricing it into the banks’ market valuation. Therefore, the banks might be particularly concerned with signalling to investors that the dividend cash flows are merely deferred until the payout ban is lifted. Hoarding the capital by restricting loan supply growth and maintaining pre-crisis target capital ratios are the ways to do it.

While any economic downturn is accompanied by uncertainty, the COVID-19 pandemic has set a record.^2^ The studies show that banks, particularly large-sized, reduce their credit supply in response to high economic and regulatory uncertainty. The recovery path of the banks’ equity prices is also extremely uncertain which makes the expected costs of replenishing capital to its pre-crisis target levels unfavourable, adding to the banks’ reluctance to make full use of the capital release.

The euro area-wide suspension of banks’ earnings distribution caused a surge in regulatory uncertainty as well and diminished the banking sector’s attractiveness for investors. As a result, this decision might incur long-term negative effects on the banks’ funding options. As the regulatory uncertainty is at the core of the problem, clear guidelines for the regulators’ decisions regarding the banking sector dividends are required. Therefore, we develop policy suggestions on the course of the supervisory actions concerning payout restrictions in pre-crisis situations analogous to the outbreak of COVID-19. We argue that the following distributional guidelines would be favourable to the banks’ valuation, funding options, and lending capacity:

1. The banks should be allowed to distribute the financial year earnings as scheduled in the form of dividends or share buybacks depending on the banks’ preferred payout method;
2. The share buybacks should be limited to the sum of the distributable earnings;
3. The coupons on the instruments qualifying as AT1 capital should not be restricted.

Alternatively, bank supervisors might consider raising the MDA trigger threshold. This decision can be reasonably justified and the mechanism is familiar to the market. As a result, the banks would be treated heterogeneously depending on their capital ratios, and those affected would still be able to distribute a portion of their earnings. Eventually, it would lead to an increase in capital ratios as banks would want to restore the managerial buffers above the expected level of the MDA trigger point.

## Bank lending in 2020 and credit guarantee schemes

### Bank lending in 2020

From the data collected by the ECB and provided through Statistical Data Warehouse (SDW), we can observe an unprecedented spike in loan volumes from monetary financial institutions (MFIs)^3^ to euro area non-financial corporations (NFCs): the total increase in loans from March to May 2020 was €245 billion or 5.5% from the total credit volume at the end of February. The peak of the aggregate credit growth, an increase of more than €120 billion, took place during March: as it is customary for the onset of economic distress, corporate borrowers drew down the available credit lines to secure their liquidity position in view of the potential disturbance in the credit markets. The resulting upswing in the risk-weighted assets (RWA) reduced the Common equity tier 1 (CET1) to RWA ratio of the euro area banking sector by 20 basis points.

**Fig. 1:**
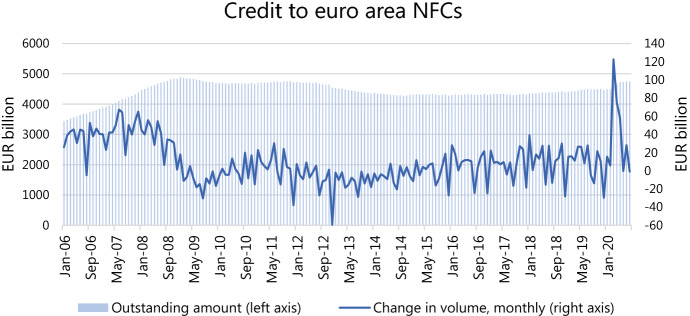
Total credit to euro area NFCs. Source: ECB Statistical Data Warehouse (SDW)

The bank lending survey (BLS) conducted by the ECB reveals that in the second quarter of 2020 the demand for loans, particularly short-term, has surged from both large non-financial firms and small and medium-sized enterprises (SMEs). The firms borrow to offset the gap in revenues and finance their everyday operations (inventories and working capital); demand for long-term investments and business expansion is depressed. The banks responded by reducing the loan application rejection rate and easing credit standards for short-term loans while tightening credit standards for long-term financing (Fig. 1).

**Fig. 2:**
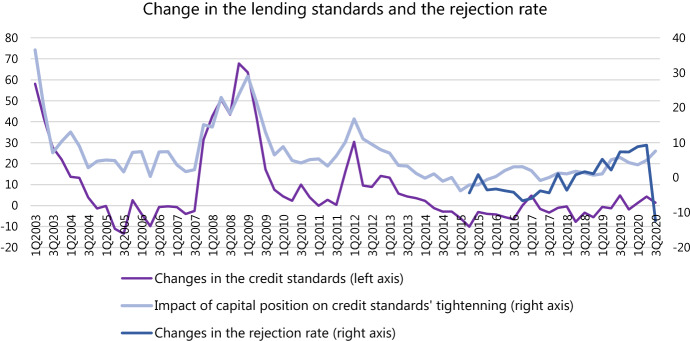
Lending standards and rejection rate. Source: The bank lending survey, SDW

The banks communicate that government loan guarantee schemes are the main factor keeping the lending standards overall unchanged and the rejection rate lower. Therefore, when asked about their expectations, the banks forecast a substantial tightening of the credit standards after the government guarantee schemes expire. By far, the key contributor to the anticipated tightening of the lending standards is the perception of risk related to exacerbation of the general economic situation and borrowers' creditworthiness. At the end of the second quarter, only 8% of banks indicated their capital position as a limiting factor for lending, which is 2% higher than in the pre-corona period. Only 2–3% of banks (a decline from 5%) reported their liquidity position to be a limiting factor (Fig. 2).

**Fig. 3:**
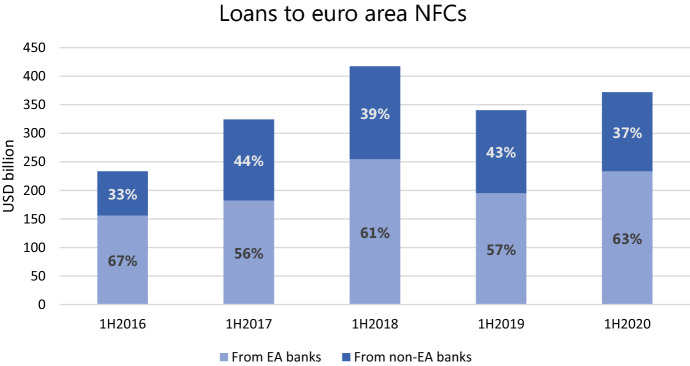
Syndicated loans to euro area NFCs. Source: WRDS − Reuters DealScan (for the observations where the allocation of the loan shares among the syndicate members is not available, a standard assumption of equal shares is made)

The data from the syndicated loans market where corporations raise a large share of their financing also indicates an increase in new lending volumes from euro area banks to the euro area non-financial sector. Figure 3 depicts the total volume of new bank loans (which it should be noted includes newly arranged credit lines and does not reflect existing credit line drawdowns) in the first six months of the years from 2016 to 2020. We can observe that in the first half of 2020, euro area corporations borrowed in the syndicated loans market 9% more than during the same period in 2019. Moreover, the share of euro area banks’ lending has increased relative to three previous years. This observation indicates the banks’ propensity to lend to large euro area companies.

**Fig. 4:**
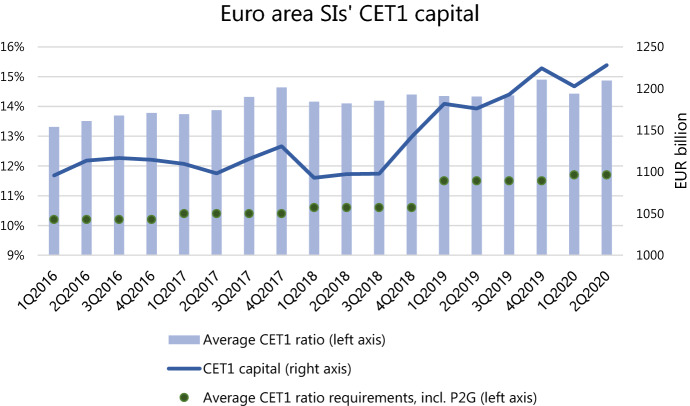
SIs’ CET1 capital. Source: Supervisory banking statistics (SDW), SREP reports

Overall, by the end of the second quarter of 2020, the total volume of loans provided by MFIs to firms and households increased by 3% relative to the start of the year (6% to firms and 1% to households)–at double the pace than during the same period in 2019.^4^ At the same time, the credit risk exposure (credit risk component of RWA) is equal to its size at the start of the year. These numbers, combined with the results of the BLS, indicate that the credit supply growth has been driven by the government guarantee schemes as the guaranteed loans obtain low to none risk-weight and, thus, do not increase the sum of RWA. A simultaneous portfolio reallocation towards safer borrowers, such as large firms with high credit ratings, would also explain the unchanged size of RWA. The significant institutions (SIs) have also restored the amount of CET1 capital during the second quarter and, as a result, their average CET1 to RWA ratio is now at its start of the year level as well (Fig. 4). Thus, we do not observe the capital buffers’ utilisation on the aggregate level. In order to make exact inference on the banks’ capital buffer utilisation or borrowers’ credit rationing, more detailed data at bank-firm level is required. It is, therefore, subject to future research.

### Credit guarantee schemes in euro area

Public guarantee schemes (PGS) became one of the key instruments in supporting the real sector in 2020. The existing PGS framework allowed for prompt response to the companies’ increased liquidity needs through expanding the guarantee programs in place.^5^ This type of credit support takes the form of government guarantees for loans issued by financial institutions to eligible companies.

The parameters of PGS introduced in response to the pandemic are heterogeneous across euro area countries in size and parameters; but the key features are largely similar. Most of the programs target primarily SMEs who tend to be credit rationed during crises more severely than larger firms. To be eligible for the program, a firm must be solvent prior to the onset of the pandemic. Although some programs offer 100% coverage, the majority of the programs split the credit risk associated with guaranteed loans between the state and the bank by offering coverage from 70 to 90%.^6^ Partial retention of the credit risk by the bank alleviates the moral hazard problem and ensures that the lender performs the borrower screening process in a duly manner.

Thus, PGS are efficient in supporting credit supply to the real sector as they alleviate the lenders’ concern regarding the credit quality of the newly issued loans which is one of the main limiting factors to the flow of credit during an economic turmoil. The expected amount of loss from a guaranteed loan is low since the state will compensate the portion of the loss corresponding to the guarantee program coverage condition. As a result, such a loan has a low risk weight when accounted for in the sum of a bank’s risk-weighted assets. Consequently, lending within PGS does not increase banks’ RWA nearly as much as it increases the total volume of their loan portfolios.

In Appendix 1, we provide a concise regression analysis to roughly assess the effect of the size of PGS relative to the country’s banking sector size on bank lending outcomes at the end of 2020. We perform the analysis at the bank level for a cross-section of the euro area banks. The results suggest that PGS were successful in stimulating credit supply as a larger span of PGS is associated with an increase in the total volume of the banks’ loan portfolios. At the same time, the average riskiness of the banks’ assets decreased in the countries where more funds were allocated for the guarantee programs, which is consistent with the portfolio expansion primarily within the PGS. The growth rate in the total sum of RWA is also lower among the banks exposed to larger guarantee program size. Finally, a standard deviation increase in the relative size of PGS is associated with a 0.5 percentage point increase in CET1, which indicates a lower capital buffer usability in the countries with accommodating credit guarantee programs.

## Bank capital regulation and target capital ratio

Banks are required to hold the amount of their own capital sufficient to absorb potential losses in their portfolios and keep the bank solvent. Bank capital requirements are estimated as a share of the bank’s RWA. Minimum capital ratio (Pillar 1) for the banks that are subject to the Basel III regulatory accord is set at 8%. The highest quality type of capital, equity and retained earnings, also called CET1, must comprise at least 4.5% of the banks' own funds. The rest of the minimum requirements can be met with the lower quality capital—Additional Tier 1 (contingent convertible bonds—CoCos^7^) and Tier 2 (subordinated debt instruments, general provisions, loss and revaluation reserves, etc., max. 2%). The higher the quality of the capital instrument, the more expensive it is for financial institutions to issue it as it implies more risks for the investors. Since 2019, the capital conservation buffer (CCoB) is de-facto another mandatory 2.5% layer of CET1 capital for all credit institutions intended to prevent any breaching of the Pillar 1 minimum requirements.

On top of the minimum requirements, prudential regulation includes additional layers of CET1 capital that differ in their key objectives. Macroprudential policy is aimed at promoting the stability of the financial system as a whole. Within the macroprudential policy framework, the capital buffers are employed in order to ensure that credit institutions are resilient to the shocks intrinsic to the respective financial system. Here, the capital-related toolkit includes the systemic risk buffer (SyRB), the higher loss absorption requirements for globally systemically important institutions (G-SII), and other systemically important institutions (O-SII). Another objective of the macroprudential regulation, for which countercyclical capital buffer (CCyB) is primarily designed, is to smooth the credit supply to the economy over the cycle, i.e., prevent excessive lending and the systemic risk build-up during the expansion phase and avoid interruption in the liquidity provision to the real economy in the time of distress. Together, the capital buffers, including CCoB, comprise the combined buffer requirements (CBR).

The microprudential framework adjusts capital according to the individual bank's risk exposure. These capital requirements, known as Pillar 2, are designed to ensure that risks not fully covered under Pillar 1 are accounted for in the total capital requirement. The banks are required to have in place the internal capital adequacy assessment process (ICAAP) that involves the use of elaborate internal models and stress tests to quantify all the risks the bank bears and estimate the amount of capital appropriate to that risk profile. Bank supervisors review ICAAP as a component of an in-depth bank evaluation (Supervisory review and evaluation process—SREP) and, as a result, the appropriate level of Pillar 2 capital add-on is determined. While Pillar 2 Requirements (P2R) are binding, Pillar 2 Guidance (P2G) is the recommended additional capital for credit institutions and breaching it does not trigger any legal or regulatory consequences.

In the euro area, whenever a capital ratio falls below the required level, an automatic restriction on the bank’s earnings distribution is triggered so that the capital is replenished with the retained portion of the earnings. However, the competent authorities can intervene at an early stage and prevent the bank’s capital from falling below the adequate level. This measure, the so-called MDA trigger, restricts payments of dividends, coupons on AT1 instruments, and employees’ variable remuneration. Therefore, the risk of MDA activation has a negative impact on banks’ market valuation and AT1 funding options.

In the early literature on banks’ capital structure, it was common to assume that banks prefer to keep their capital ratios close to the requirements level because equity funding is costly, while the implicit government’s guarantees reduce the risk of bank debt making this source of funding cheaper for banks. However, in practice, we observe substantial variation in the size of banks' capital buffers above the combined regulatory requirements. Therefore, Gropp and Heider (2010) study this problem in the sample of publicly traded US and EU banks during the period 1991–2004 and find that capital regulation is indeed of second-order importance in determining the capital structure. They uncover that banks maintain stable leverage ratios over time at the level specific to each bank. Certain bank characteristics, such as size and risk, do explain the variation in leverage between the banks. However, 92% of the variation is driven by bank fixed effects which means that the bank's capital structure cannot be explained by its observable time-varying characteristics. Rather, there are relatively unchanging managerial and shareholders' preferences and the choice of a business model that determine banks’ target capital structure.

Berger et al. (2008) also conclude that banks have individual target capital ratios above the minimum regulatory requirements, actively manage their capital structure, and promptly adjust it towards their targets. Empirical evidence indicates that banks target the managerial buffer above the regulatory minima rather than the total level of the capital ratio: Bridges et al. (2014) find that, in response to an increase in capital requirements, UK banks gradually rebuild the buffers that they initially held. Even well-capitalised banks are responsive to changes in capital requirements (De Jonghe et al., 2020).^8^ The reasons for banks to maintain a cushion of excess capital include the reduced risk of supervisory interventions, higher credit rating and, hence, lower funding costs, and flexibility to take advantage of potential investment opportunities.

When capital requirements increase (decrease), the capital ratio (equity over RWA) can be adjusted by (i) Issuing new equity (distributing dividends and/or share repurchase), (ii) Reallocating loan portfolio towards safer (riskier) assets with lower (higher) risk weights, (iii) Curtailing (expanding) lending. The studies show that in practice, when banks are adapting to changes in capital requirements, they do it primarily by adjusting the loan volumes to corporate borrowers. Gropp et al. (2019) show that large European banks react to the increase in capital requirements by reducing lending to corporate and, to a lesser extent, retail customers. In Imbierowicz et al. (2018),^9^ Danish banks react to a decrease in the requirements by expanding the corporate loans portfolio as well and, additionally, mildly reducing their equity holdings, presumably through dividend payments or share repurchase.

## The measures undertaken by the ECB and national authorities

On the 12th of March 2020, the ECB announced a set of regulatory decisions aiming to facilitate stable credit supply from the banking sector to the coronavirus-afflicted economy.^10^ The package of measures included the following:

- The release of the Pillar 2 Guidance capital requirements
- The release of the capital conservation buffer
- The request to suspend dividend payments and share buybacks (later that month)
- The release of the liquidity buffer (liquidity coverage ratio – LCR)
- The adjustments to the structure of the capital for Pillar 2 Requirements

The ECB provided an estimation of the amount of CET1 capital released by these measures—€120 billion were made available for loss absorption and lending. This estimate accounts for the decisions related to P2G and P2R for significant and less significant institutions, that together hold more than 80% of banking assets in the euro area and does not account for the effect of CCoB release.^11^ The ECB does not include CCoB release in the reported estimate probably assuming that the banks will be particularly reluctant to go below this last line of defence before Pillar 1 minimum capital requirements. Excluding CCoB from the estimations might also signal that while the regulator encourages the banks to step up and use other buffers release for expanding their loan portfolios, it expects banks to preserve CCoB for the anticipated loss absorption.

The adjustment to the structure of capital for P2R constitutes the replacement of 100% CET1 requirement with 56.25% CET1, 18.75% Additional Tier 1, and 25% Tier 2 types of capital. However, banks might be reluctant to promptly make use of the P2R adjustments as the market for AT1 and Tier 2 is currently tense and unavailable for riskier issuers. For example, the yields for CoCo bonds are elevated, and new issues were severely depressed from March to May. The average size of P2R requirements for SREP banks in 2020 is 2,1%. Therefore, the adjustment to this requirement releases 0,9 pp of CET1 over RWA ratio, or €75 billion. Presumably accounting for the limitations of rapid replacements of CET1 with AT1 and Tier 2, the ECB estimated the effect of P2R adjustments as equal to €30 billion. We rely on this estimate in our assessment.

Following the relief of the requirements announced by the ECB, the national macroprudential authorities of the euro area countries released countercyclical capital buffers and reduced or released systemic risk buffers (SyRB and O-SII), which additionally freed up over €20 billion. Together, the released capital buffers sum up to 1.7 pp of CET1 over RWA (see Table 1).

**Table 1:** CET1 capital released in 1H2020

	Billion, EUR	Ratio to RWA
Total CET1 capital	1222.4*	14.9%*
Pillar 2 Guidance	90.0	1.1%
Pillar 2 Requirements	30.0	0.4%
CCyB	13.7	0.2%
SyRB	7.5	0.1%
O–SII buffers	0.6	0.0%
CCoB	205.5	2.5%
Total without CCoB	**141.8**	**1.7%**
Total with CCoB	347.3	4.2%

Bold values refer to the figures mentioned in the text

Source: Statistical Data Warehouse. *Data on 31.12.2019; the ECB’s summary of measures taken by national authorities (version on 8 July 2020)

*CET1* common equity tier 1, *CCyB* countercyclical buffer, *SyRB* systemic risk buffer, *O-SII* other systemically important institutions, *CCoB* capital conservation buffer

It was the end of July when the ECB clarified the timeline for reverting to the regular capital and liquidity requirements regime.^12^ The time gap between announcing the policy decision and its expiration date has contributed to the uncertainty perceived by the market participants. The ECB announced that it would not require banks to start replenishing their capital and liquidity buffers before the peak in capital depletion is reached and they will be allowed to operate below P2G and CBR until at least the end of 2022, and below the LCR until at least the end of 2021.

## The expected impact on the credit supply

### The range for the impact

The unprecedented nature of the current economic situation–the exogeneity of the problem both to the financial and real sectors–complicates the estimation of the expected effect of the capital buffers release. The studies investigating the effect of capital regulation on lending do it either in the context of the GFC, when the credit supply shock originated in the financial system, or in the context of a change (mostly increase) of the capital requirements for banks during non-crisis periods. The early stage of the coronavirus crisis differs from the GFC set-up because the banks’ lending capacity has not been damaged by funding disruptions or portfolio defaults. It also differs from the requirements change set-up because of the hard hit experienced by the real sector and the extreme uncertainty associated with all the components of the banks’ business environment.

To approximately evaluate the expected magnitude of the March 2020 capital release impact, we have selected a number of papers that study the effect of the change in capital requirements or the effect of the level of the requirements on bank credit supply to the real economy. The papers are listed in Table 2 in descending order of their relevance to the current situation in the euro area banking sector. The first two papers, Mésonnier and Monks (2015) and Berrospide and Edge (2019), examine the effect of the increase in capital requirements for locally large banking groups in Europe and the USA. Considering that banks respond to the changes in capital requirements in any direction by predominantly adjusting their corporate loans portfolios, it is reasonable to assume that this response is symmetric to the increases and reductions of the capital requirements. Therefore, the range of 1.2–1.5% growth in credit supply per 1 percentage point of capital requirements reduction is our baseline prediction. For a 1.7-pp capital release it translates into a 2.0–2.6% increase in lending to the real economy. If we extend the span of literature by including the recent papers that focus on the relationship between the total level of capital requirements (rather than the change in requirements) and bank lending, then the forecast range expands to 0.9–6.6%.

**Table 2:** The estimation of the expected impact of the capital release on commercial lending based on the empirical literature

Paper	Tool	Geography	Period	Object of examination	Impact of 1 pp change in capital requirements. annualised	Impact of 1.7 pp capital release (without CCoB)
Mésonnier and Monks (2015)	EBA capital exercise	Europe	2011–2012	Change in CR*	1.2	2.0
Berrospide and Edge (2019)	Stress–test to CET1 ratio	USA	2012–2016	Change in CR	1.5**	2.6
Fraisse. Lé. and Thesmar (2019)	Heterogeneous time–varying CR	France	2008–2011	Level of CR	2.1	3.6
De Jonghe, Dewachter, and Ongena (2020)	Pillar 2 (P2R)	Belgium	2013–2015	Level of CR	0.5	0.9
Bridges et al. (2014)	Heterogeneous time–varying CR	UK	1990–2011	Level of CR	3.9	6.6

*CR Capital requirements

**The upper range for our baseline prediction coincides with the estimated effect in Altavilla et al. (2020)

It is, however, important to note that in all the studies, the inference about the magnitude of the regulatory impact is made based on the comparison of lending by the “affected” group of banks to that of the “control” or “less affected” group of banks. This means that the results can only be interpreted as the deviation of the lending behaviour by the affected banks from how they would have behaved in the absence of any regulatory changes. In the context of our analysis, it means that the actual credit supply growth can be lower or higher than 2.0–2.6%, but that is how much the capital buffer release is expected to contribute to the credit supply growth within one year of the decision being announced by the ECB.

We expect the capital relief to have an immediate effect on the banks’ whose capital ratios were close to the binding regulatory minima as their lending capacity was otherwise modest. Lowering the capital threshold by more than 4 pp has removed the banks’ incentive to deleverage in the middle of economic turmoil and gave them the flexibility to roll over and provide additional loans to their borrowers, thus preventing early loan delinquencies in their portfolios. Going forward, these banks will have the capacity to absorb losses while maintaining normal business operations.^13^ Some of the banks with riskier business models are more likely to make the full use of the released capital in order to take advantage of the safety net of the explicit and implicit government guarantees for the banking system. This prediction is motivated by moral hazard incentives widely assumed for distressed credit institutions: if a bank has a high probability of going bankrupt, taking on more leverage is consistent with the shareholders’ value maximisation as the shareholders do not bear the full costs associated with the risk-taking (their losses are limited by their stake in the equity which they lose in case of bankruptcy anyway), but receive all of the benefits if the adverse scenario does not materialise. The recent study by Ben-David et al., (2020), however, does not find this strategy to be prevalent among distressed banks during the GFC.

A 2.0–2.6% increase in bank loan volume provided by significant and less significant institutions, that together hold 80% of banking assets in the euro area is approximately €72–93 billion of new loans during the twelve months after the capital relief announcement. The magnitude of the impact of the capital availability will magnify over time if the crisis exacerbates and the losses start accumulating on the banks’ balance sheets: Jimenez et al. (2017) demonstrate, among other things, how the effect of the release of Tier 2 capital on lending accumulates over time, ranging from 9 to 32% depending on the estimation method, two years after the start of the GFC. The large distance between the crisis and non-crisis capital impact coefficients indicates that capital buffers facilitate lending primarily through loss absorption. Hence, the availability of the bank capital will become increasingly important as the recession unfolds.

At the moment, we observe that the actual growth of aggregate credit supply exceeds the range of our predicted effect (Sect. 2). Currently, the two driving forces of the loan volume growth are the drawdowns of pre-committed credit lines, lending to non-risky borrowers, and government loan guarantee schemes. At the same time, banks might be reluctant to extend credit to the borrowers that have a non-zero risk of default and do not benefit from the guarantee schemes. In the next subsection, we discuss in details the factors that motivate banks to abstain from the capital buffers utilisation.

### The limiting factors and policy suggestions

Naturally, there are many factors banks have to consider when making the decision about expanding their loan portfolio volume, and regulatory constraints are only some of them. Since the beginning of the corona-crisis, the ECB has relaxed funding, liquidity, and capital constraints, however, market discipline considerations remain in place and can confine the credit supply. Market discipline generally refers to the influence that the market participants’ reaction has on a financial institution’s decision making. Market participants, such as shareholders, large depositors,^14^ and other creditors, continuously monitor a bank. When the bank acts in a way that increases the risks associated with its business, market participants react by selling the financial instruments issued by the bank which reduces these instruments’ valuation such that it reflects the increased risk. As a result, the bank’s funding costs grow while the shareholders’ wealth and, hence, the bank managers’ remuneration are negatively affected.

Since the beginning of 2020, European banks’ market valuation experienced a sharp decline: the difference between the highest and the lowest values of the EURO STOXX Banks Price Index that year was more than 50%. During March, as the global epidemiologic and market situation deteriorated, the bank stock index lost 35% (the broad-based index EURO STOXX 50 decreased by 16%, see Appendix 2) revealing investors’ concerns about the banking sector risks and profitability.

The ECB’s recommendation to suspend share buybacks and dividends for the financial years 2019 and 2020 was issued on the 27th of March.^15^ It was followed by a drop in the EURO STOXX Banks Price Index of 11% over two trading days following the announcement of the ECB recommendation.^16^ The payout moratorium was intended to preserve bank capital and liquidity for lending and loss absorption and reduce the amount of public funding support in case of a bank bailout.

However, when it comes to its effect on bank lending, the consequences of the payout ban can be twofold. On the one hand, retained dividends are an additional layer of CET1 capital that gives the banks room to absorb losses and expand their loan portfolios before breaching the regulatory requirements. Moreover, this regulatory decision is justified from a historical perspective. Multiple studies^17^ document that at the onset of financial crises banks tend to increase their earnings distribution and, thus, remunerate shareholders at suboptimally high levels shifting the risks from the equity holders to the depositors and debt holders. The motivation behind such a distribution policy is to smooth the shareholders’ cash flow over the economic cycle as well as to signal to the market the bank’s confidence in its own financial stability and resilience to the crisis.^18^ During the GFC, under the Basel II framework,^19^ this costly signalling in combination with accumulating losses eroded banks’ capital and impaired their lending capacity. Thus, from this point of view the retained dividends should have a positive impact on bank lending.

On the other hand, the sharp negative market reaction to the payout ban could put the market discipline considerations to the fore in banks’ decision making. Dividend flow is an important determinant of banks’ market value. One of the most popular methodologies to estimate bank valuation is Dividend Discount Model (DDM). DDM postulates that the value of a company is equal to the sum of the discounted dividend payments. An unexpected restriction on dividend payout not only nullifies the next dividend payment but also increases the rate at which the future dividend cash flow is discounted since it is now less certain. An elevated cash flow risk reduces the price at which banks’ stocks are traded in the market. As a result, if a bank wants to raise a certain amount of additional equity capital, at lower prices it will have to issue a higher number of new stocks and, thus, dilute the share and the rights of existing shareholders. In other words, lower market valuation implies a negative outlook on banks’ funding options.

Apart from impeding dividend cash flow to the shareholders, the payout ban caused a surge in economic policy uncertainty. Before March 2020, there was no precedent or regulation alerting the investors to the fact that their dividend cash flow can be redirected towards the banks’ portfolio expansion in the face of an economic downturn. Therefore, the market is now pricing in the new source of uncertainty stemming from this and other potential regulator’s decisions into the banks’ market capitalisation. Moreover, the payout ban suggests that the ECB might consider the current level of capitalization in the banking sector insufficient. Hence, the banks might face an increase in capital requirement in the foreseeable future.

In general, economic policy uncertainty and regulatory uncertainty have a significant negative effect on bank credit growth (Bordo et al., 2016; Gissler et al., 2016). This negative effect of uncertainty is particularly strong for larger banks, such as those supervised by the ECB directly, as they are subject to greater regulatory scrutiny (Bordo et al., 2016; Hu and Gong 2019). Lending outside the credit guarantee programs under economic uncertainty exposes banks to higher credit risk that increases their RWA and, as a result, reduces the regulatory capital ratios. In combination with the regulatory uncertainty regarding the capital requirements and the uncertainty regarding the payout restrictions that negatively affect the expected funding costs, the value-maximising behaviour for an individual bank is to confine corporate lending.

Furthermore, in an attempt to mitigate the negative impact of the regulatory shock on their valuation, well-capitalised banks might be particularly concerned with signalling to the investors that the dividend cash flows are merely deferred, not jeopardised by the current situation, and will be restored as soon as the payout ban is lifted. This signal can be communicated in the form of preserving the managerial capital buffer at its pre-crisis level and avoiding additional risk-taking.

These considerations emphasise the importance of developing clear guidelines for payout restrictions to mitigate the negative effect of the uncertainty surge on credit growth. In our opinion, the regulatory framework should explicitly state the course of the supervisory actions concerning payout restrictions in pre-crisis situations analogous to the outbreak of COVID-19. Clear guidelines would allow the market to price the risks of the bank financial instruments’ cash flows correctly and, thus, avoid overpricing these risks and abate the instruments’ price volatility. We would also recommend less stringent restrictions than those introduced at the onset of the pandemic, as dividends are an important determinant of banks’ valuation. In the EU bank bailout practice, such stringent restrictions have not always been imposed on banks despite being efficient for the banks’ speed of financial recovery and ex-ante incentives (Berger et al., 2020). We, therefore, would recommend the following distributional guidelines for banks when an economic or financial turmoil is anticipated:

1. The banks should be allowed to distribute the financial year earnings as scheduled in the form of dividends or share buybacks depending on the banks’ preferred payout method;
2. The share buybacks should be limited to the sum of the distributable earnings;
3. The coupons on the instruments qualifying as AT1 capital should not be restricted.

The first point is required to restore the market’s confidence in the stability of dividend flow which in turn should reduce the cost of raising equity for banks in crisis and post-crisis periods and make them less stingy with their capital. The share buybacks restriction is aimed at addressing the regulators’ concern about the banks dissipating their capital. Indeed, the banks might find it value-maximising to repurchase the stocks after they experience a dramatic price drop and are undervalued from the managers’ point of view^20^. The repurchase would signal the managers’ confidence in the bank’s future performance and increase earnings per share for the remaining shareholders. At the same time, the banks might underestimate the future shock to their portfolios and end up with the capital and liquidity levels insufficient to withstand the crisis.

AT1 bonds were introduced after the GFC as a cheaper source of loss-absorbing capital for banks. By design of the instrument, the CoCos’ holders bear the same risk of default as shareholders but do not benefit from the bank’s stocks’ upside potential. For CoCos to remain a cheaper source of bank funding than equity, these instruments should have lower cash flow risks than stocks. In other words, the attractiveness of the instruments for the investors and, hence, the existence of the market for CoCos hinges on stable and predictable coupon flow. Therefore, we recommend not to restrict AT1 coupon payments beyond the cases considered in the current supervisory framework (MDA trigger).

When dealing with strong shocks, in the future, bank supervisors might additionally consider raising the MDA trigger threshold. This decision can be reasonably justified as the bank stress tests might not fully account for such rare events; moreover, some shocks are difficult to model within the stress test framework, e.g. shocks stemming from the climate change. As a result, the risk of breaching minimum capital requirements level by lower capitalised banks turns out to be higher than expected. If the suggested adjustment is clearly articulated, this would be a familiar mechanism to the market, which is already accounted for in the banks’ capital planning and priced by the market according to its current application parameters. As a result, the banks would be treated heterogeneously depending on their capital ratios, and those affected would still be able to distribute a portion of their earnings. Eventually, it would lead to an increase in capital ratios as banks would want to restore the managerial buffers above the expected level of the MDA trigger point.

Interestingly, under the payout ban, some banks opted for stock dividends, i.e., shareholder remuneration in a form of distributing newly issued stocks proportional to their stake in the company. For example, Banco Santander S.A. did so in November 2020. Such a form of shareholder remuneration does not weaken bank capital and liquidity position. However, it can ease the pressure on the bank management through behavioral mechanisms. First, it gives the shareholders an opportunity to sell the newly issued shares and receive cash while maintaining the initial nominal amount of shares. Secondly, companies often aspire to keep the sum of a dividend payment per share constant. Hence, more shares can imply the management’s intention to distribute more earnings in the form of dividends in the future.

Another dominant challenge lenders are currently facing is the extreme degree of uncertainty prevailing in all the components of the business environment. Uncertainty is one of the key factors behind the decline in loan supply during the period of economic distress. A decision about granting a loan is akin to a decision “to invest”. In the presence of high uncertainty, it is rational for economic agents to defer investments. This conclusion stems from the option-based approach to corporate finance, where investment decisions are viewed as real options. The “option to wait” then has a value that increases with uncertainty, and making an investment extinguishes that option, destroying its value for the firm. So, the value of the option to wait is one of the opportunity costs of the investment (Kandel and Pearson 2002). Bloom et al. (2007) show that higher uncertainty reduces the responsiveness of corporate investment to demand shocks and policy stimulus. In other words, the entities become more cautious in their investment behaviour in the presence of high uncertainty.

The literature shows that banks exhibit similar investment behaviour under high uncertainty. Numerous studies find that banks respond to the aggregate uncertainty (Alessandri and Bottero 2020), economic policy or regulatory uncertainty (Bordo et al., 2016; Gissler et al., 2016), and banking sector uncertainty (Buch et al., 2015) by reducing credit supply. The studies are also in agreement that better capitalised banks and banks with larger liquidity buffers contract lending by less. Once again, Bordo et al., (2016) find that the banks respond by primarily adjusting their corporate loans portfolios and reducing the maturities of the loans. Lending long-term in such conditions exposes banks to higher credit, liquidity, and funding risks. The recovery path of the banks’ equity prices is also extremely uncertain which makes the expected costs of replenishing capital within the timeline that will be specified by the ECB unfavourable, adding to the banks’ reluctance to make full use of the capital release.

## Conclusion

In this article, we describe the lending conditions for the euro area borrowers at the onset of the COVID-19 pandemic and discuss the regulatory actions directed at stimulating bank credit supply to the real sector. We document that the regulatory measures were successful in removing liquidity- and capital-related constraints to the credit supply. In a supplementary analysis, we demonstrate that credit guarantee schemes activated in response to the pandemic contributed substantially to the growth in banks’ lending volumes. At the same time, the state guarantees decrease the banks’ credit risk exposure, thus, reducing the extent of the banks’ capital buffers utilization.

Further, we argue that the sudden dividend distributing restrictions caused a surge in regulatory uncertainty for the banks and their shareholders. The dividend ban undermined banks’ market valuation raising the expected funding costs and contributing to the banks’ reluctance to make use of the capital buffers. We argue that, to mitigate the effect of the regulatory uncertainty shock, clear policy guidelines regarding the introduction of payout restrictions in the future are required. Finally, we develop suggestions for apt payout restrictions.

## Figures and Tables

**Fig. 5:**
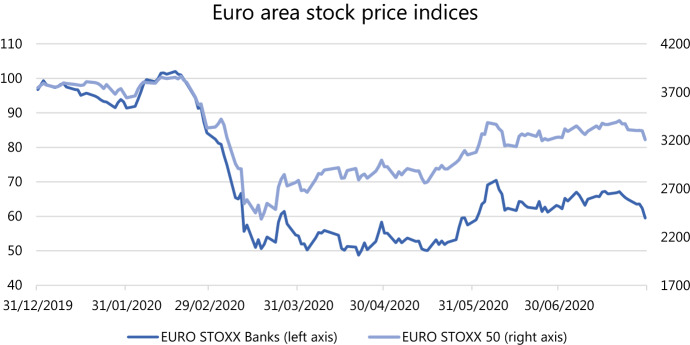
Euro area stock price indices. Source: https://qontigo.com/

**Fig. 6:**
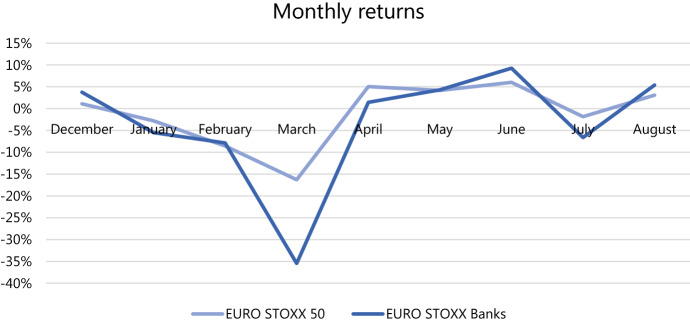
Monthly returns on euro area stock price indices. Source: https://qontigo.com/, authors’ calculations

**Fig. 7:**
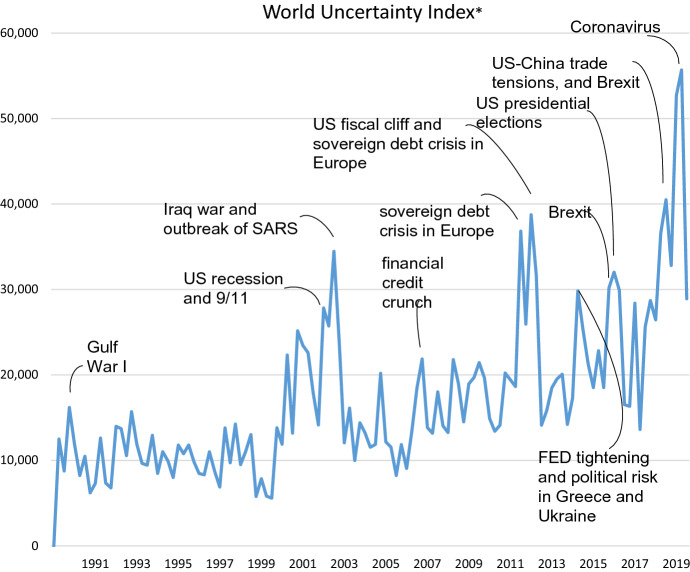
World Uncertainty Index. Source: Ahir et al., (2018), Stanford mimeo

## Appendix 1

In this section, we present the result of the regression analysis that investigates the effect of public guarantee schemes (PGS) introduced in response to the coronavirus pandemic in the euro area on bank lending and risk measures. We observe that PGS had a positive effect on the size of the banks’ loan portfolios while it is associated with a decrease in the riskiness of banks’ assets.

## Data

For the analysis, we use consolidated end-of-the-year financial statements of euro area banks from FitchConnect. We take the data on the volume of PGS by country from Budnik et al. (2021). We supplement this data with GDP per capita from the World Bank and bank sector size by country from thebanks.eu website.

## Model and variables

We run the following cross-section model at bank-level:

1 $$\Delta Y_{bc} = \, \alpha \, + \, \beta PGS_{c,2020} + \, \gamma {\prime }X_{b,2019} + \, \eta GDP_{c} + \, \varepsilon_{bc}$$

where ΔY_bc_ is a set of dependent variables that measure the change in lending outcomes at bank *b* from country *c* from the end of 2019 to the end of 2020. β measures how much the size of the guarantee program introduced in country *c* during 2020 contributed to the change in those lending outcomes. We scale the size of PGS (in bln EUR) by the size of the banking sector in the respective country. γ’X_b,2019_ is a vector of bank b balance sheet controls such as bank size, profitability, liquidity, leverage, and share of deposit funding at the start of 2020. To capture the difference between economic development between the countries we include in the model the log of GDP per capita.

After consistently constructing the set of dependent and control variables, we obtain a sample of 708 banks from 18 countries. The descriptive statistics of the variables is presented in Table

**Table 3:** Descriptive statistics

Variables	(1)	(2)	(3)	(4)	(5)
	N	Mean	sd	Min	Max
Volume of PGS over Bank Sector Size	708	7.615	4.143	0	10.66
Log change in total loans	708	0.0548	0.0990	− 0.383	0.716
Log change in net loans	708	0.0558	0.1000	− 0.421	0.693
Log change in non–mortgage loans	695	0.0611	0.187	− 0.693	0.970
Log change in total RWA	708	0.0272	0.0921	− 0.241	0.423
Log change in credit risk part of RWA	238	0.0256	0.123	− 0.327	0.362
Change in RWA/Total Assets (pp)	708	− 3.018	4.396	− 14.07	12.85
Change in CET1 ratio (pp)	708	0.210	2.191	− 13.32	8.090
ROA (Net Income over Total Assets)	708	0.361	0.685	− 2.519	7.565
Liquidity (LIQUID ASSETS OVER TOTAL ASSETS)	708	4.628	6.682	0	49.29
Leverage (common equity over total assets)	708	0.100	0.0510	0.0139	0.772
Ln total assets	708	21.46	2.065	17.41	27.57
Share of deposit funding	708	0.685	0.178	0.0309	0.902
Ln GDP per capita	708	10.63	0.243	9.787	11.65

^3^.

## Results

The estimation results are presented in Table

**Table 4:** Effect of PGS on bank lending in the euro area in 2020

Variables	(1)	(2)	(3)	(4)	(5)	(6)	(7)
	Log change in total loans	Log change in net loans	Log change in non–mortgage loans	Change in RWA/Total assets (pp)	Log change in Total RWA	Log change in credit risk part of RWA	Change in CET1 ratio (pp)
Volume of PGS over Bank Sector Size (standardized)	0.0096***	0.0103***	0.0257**	− 1.0331***	− 0.0111***	− 0.0343***	0.4802***
	(0.003)	(0.004)	(0.011)	(0.175)	(0.004)	(0.008)	(0.107)
Ln Total Assets	− 0.0106***	− 0.0110***	− 0.0123**	− 0.3974***	− 0.0098***	− 0.0167***	0.1501***
	(0.003)	(0.003)	(0.005)	(0.090)	(0.002)	(0.004)	(0.049)
Deposit Funding	− 0.0041	− 0.0065	− 0.0157	0.9267	0.0812***	0.0484	− 0.6301
	(0.023)	(0.024)	(0.042)	(1.109)	(0.021)	(0.041)	(0.526)
Leverage	− 0.4158**	− 0.4357**	− 0.6174**	2.8868	− 0.0264	− 0.2764	− 12.5282***
	(0.200)	(0.202)	(0.279)	(4.097)	(0.128)	(0.219)	(2.767)
ROA	0.0337**	0.0316**	0.0438*	0.1203	0.0439***	0.0450***	0.2529
	(0.014)	(0.014)	(0.025)	(0.408)	(0.008)	(0.011)	(0.232)
Liquidity	0.0006	0.0007	0.0016	0.0589*	− 0.0013**	− 0.0010	0.0131
	(0.001)	(0.001)	(0.002)	(0.035)	(0.001)	(0.001)	(0.016)
Ln GDP per capita	− 0.0090	− 0.0200	− 0.0135	4.4151***	0.0544***	0.0549**	− 1.4119***
	(0.019)	(0.019)	(0.034)	(0.859)	(0.016)	(0.026)	(0.402)
Constant	0.4059**	0.5363***	0.5164	− 42.5452***	− 0.4030**	− 0.2265	13.4802***
	(0.197)	(0.197)	(0.321)	(9.418)	(0.175)	(0.269)	(4.503)
Observations	708	708	695	708	708	238	708
R–squared	0.082	0.087	0.047	0.117	0.201	0.341	0.167

This table examines the effect of public (credit) guarantee schemes (PGS) activated in response to the COVID–19 pandemic in the euro area on the banks’ lending volumes and risk exposure measures. The variable of interest is the volume of PGS introduced in each country normalized by the size of the banking sector in the respective country. This variable is standardized such that it has zero mean and unit variance. and we can interpret point estimates as the effect of one standard deviation change. Th dependent variables are the change in variables between the end of 2020 and the end of 2019. The control variables are the balance sheet parameters at the end of 2019 and natural logarithm of GDP per capita in 2019. Robust standard errors in parentheses. *** *p* < 0.01. ** *p* < 0.05. * *p* < 0.1

^4^. The variable of interest, the volume of PGS over bank sector size in country *c*, is standardized such that it has zero mean and unit variance, and we can interpret point estimates as the effect of one standard deviation change.

In columns 1 and 2, the dependent variables are the changes in the volume of banks’ loan portfolios: volume of total loans issued and volume of loans net of expected loan losses, respectively. We can observe that a standard deviation change in the relative size of PGS is associated with a 1-percent increase in the volume of banks’ loan portfolios. In column 3, the dependent variable is the volume of loans net of residential mortgages^21^ such that commercial loans constitute a larger share of the loan amount. We can observe that the effect of PGS is higher for such a loan portfolio; namely, 2.6 percent.

Next, we investigate the effect of PGS on an array of bank portfolio risk measures. In column 4, the dependent variable is the change in the ratio of total risk-weighted assets (RWA) over total assets. Hence, the point estimate of interest is interpreted as a 1 percentage point decrease in bank assets’ riskiness in response to a higher span of PGS. In columns 5 and 6, the dependent variables are the change in the sum of total RWA and the credit risk part of RWA.^22^ From these specifications, we infer that the state guarantees are associated with a decrease in the sum of bank RWA, particularly, through a decrease in the banks’ credit risk. Finally, a standard deviation higher value of PGS is associated with a 0.5 percentage point increase in the CET1 (common equity tier 1 capital over RWA) ratio at the end of 2020.

## Appendix 2

EURO STOXX Banks Index comprises 22 listed euro area banking groups.

EURO STOXX 50 Index comprises 50 listed euro area firms from different sectors, including 4 banks.

See Figs. 5 and 66.

## Appendix 3

The World Uncertainty Index developed in Ahir et al. (2018) is constructed based on the quarterly Economist Intelligence Unit country reports for 143 countries. The index captures uncertainty related to economic and political developments regarding both short-term and long-term concerns.

See Fig. 7.

## Footnotes

1 Not accounting for the 2.5 pp CCoB release.

2 See Appendix 3 for the World Uncertainty Index.

3 Here and further MFIs excluding the ECB and the national central banks.

4 At the end of August 2020, the increase is loan volumes is as follows: 6.4% to firms, 1.6% to households, 3.6% total.

5 See SAFE Finance Blog article “Are credit guarantee programs the right medicine for small businesses?” by Thomas Mosk for a discussion of the guarantee schemes’ unintended effects.

6 For more details about PGS see Budnik et al. (2021) or the EBA overview of public guarantee schemes in response to the COVID-19 pandemic.

7 In euro area, for CoCos to be qualified as AT1 capital they must be perpetual bonds with discretionary coupons that are automatically converted into equity if a bank’s CET1 ratio falls to 5.125%.

8 The paper observes that Belgian banks with managerial buffers larger than 3% respond to higher capital requirements by a reduction in lending while the banks with small buffers prefer to raise new equity.

9 To our knowledge, this is the only paper that considers a decrease in CET1 requirements in the analysis. We, however, do not use the estimates from this paper for the impact forecasting as the magnitude of the effect in the study is driven by smaller banks.

10 See ECB press release, 12 March 2020.

11 More details about this estimate were provided in the May 2020 issue of the ECB’s Financial Stability Review, Chart 5.1.

12 See ECB press release, 28 July 2020.

13 Gropp and Heider (2010).

14 Iyer, Puri, and Ryan (2013).

15 First and foremost, the ECB’s recommendations apply to the largest euro area banking groups that are supervised directly by the ECB. Although, the national authorities normally relay such recommendation to the smaller banks under their supervision, they are at liberty to make decision on case by case basis. Thus, for example, German financial regulator BaFin has lifted the dividend ban for some cooperative and savings banks in September 2020, before the ECB allowed moderte payout in December the same year. (Article here).

16 The broad-based index EURO STOXX 50 fell by 3% over the same period.

17 Acharya et al. (2011), Saunders and Wilson (2020), Muñoz (2020).

18 Kauko (2012) argues that while paying dividends in the bad state is costly for a bank, not doing so could trigger a deposit run. Forti and Schiozer (2015) find that during the acute period of the GFC, many Brazilian banks increased their dividends to signal liquidity and asset quality to institutional investors in an attempt to prevent bank runs.

19 Basel II prescribed banks to hold a minimum of 2% of common equity against risk-weighted assets.

20 Stephens and Weisbach (1998) show that share repurchases are negatively related to prior stock price performance, suggesting that firms increase their purchasing depending on the degree of perceived undervaluation.
